# Tools for evaluating team performance in simulation-based training

**DOI:** 10.4103/0974-2700.70746

**Published:** 2010

**Authors:** Michael A Rosen, Sallie J Weaver, Elizabeth H Lazzara, Eduardo Salas, Teresa Wu, Salvatore Silvestri, Nicola Schiebel, Sandra Almeida, Heidi B King

**Affiliations:** Department of Psychology & Institute for Simulation and Training, University of Central Florida, USA; 1 Maricopa Integrated Health System, Rochester, MN, USA; 2 Orlando Health, Rochester, MN, USA; 3 Mayo Clinic, Rochester, MN, USA; 4 Healthcare Consulting, LLC, USA; 5 Office of the Assistant Secretary of Defense (Health Affairs) TRICARE Management Activity, USA

**Keywords:** Simulation-based team training, simulation, team training, team performance measurement, team evaluation, team performance

## Abstract

Teamwork training constitutes one of the core approaches for moving healthcare systems toward increased levels of quality and safety, and simulation provides a powerful method of delivering this training, especially for face-paced and dynamic specialty areas such as Emergency Medicine. Team performance measurement and evaluation plays an integral role in ensuring that simulation-based training for teams (SBTT) is systematic and effective. However, this component of SBTT systems is overlooked frequently. This article addresses this gap by providing a review and practical introduction to the process of developing and implementing evaluation systems in SBTT. First, an overview of team performance evaluation is provided. Second, best practices for measuring team performance in simulation are reviewed. Third, some of the prominent measurement tools in the literature are summarized and discussed relative to the best practices. Subsequently, implications of the review are discussed for the practice of training teamwork in Emergency Medicine.

## INTRODUCTION

Healthcare is in the midst of a variety of transformations. New technologies are being introduced that change the way care is provided (e.g., robotic surgery) and managed (e.g., electronic health records), as well as the way care providers are educated and trained (i.e., simulation). While simulation-based training (SBT) has been a mainstay educational method in other domains for decades, only recently has its potential been widely acknowledged in healthcare. This adoption of new technologies into care delivery processes and into the development and education of personnel is accompanied by new social and organizational structures as well, most notably teamwork and team training. Good measurement practices go hand in hand with effective design of practice-based learning activities; however, team performance measurement is an often overlooked aspect of designing SBT for teams (SBTT).

This article focuses on the intersection of these two new areas of interest in healthcare: SBT and teamwork training. More specifically, the purpose of this article is to provide a review of the state of the science and practice of team performance measurement in SBT. To this end, four main goals are addressed. First, we provide a review of the fundamental concepts of team performance measurement and evaluation within the context of SBT. Second, we provide a set of best practices rooted in the science of teams, training, and performance measurement to guide the development or selection and modification of measurement tools for a specific context. Third, we review illustrative examples of tools currently available in the healthcare literature. Fourth, we identify gaps in the current state of the literature and discuss future needs.

## OVERVIEW OF TEAM PERFORMANCE EVALUATION

This section reviews key concepts and terminology that provide the groundwork for understanding team performance measurement and evaluation in SBT. Specifically, issues of purpose (i.e., why evaluate team performance?), content (i.e., what to evaluate?), and method (i.e., how to evaluate?) are provided below. First, some basic definitions are provided.

While *performance* refers to the actual behaviors enacted by a team (or a system of teams), *effectiveness* refers to the evaluation of the results of performance; that is, the degree to which these behaviors satisfy team, organizational, and/or other super-ordinate goals.[1] Through this lens, the term team performance embodies teamwork—the behavioral, cognitive, and affective processes teams engage in order to coordinate their interactions toward shared goals.[2,3] Team performance evaluation can, thus, be defined as the application of standardized diagnostic measurement tools to assess the behaviors, cognitions, and attitudes enacted by team members in relation to clearly operationalized criteria. As such, evaluation is designed to provide information not only on *what* outcomes the team achieved, but also *how* they reached these outcomes.[4] A scientifically rooted and practically meaningful approach to SBTT is built upon a foundation of diagnostic evaluation.

### Why evaluate team performance?

Team performance evaluation serves several purposes within SBT for teams. Uses of team performance in evaluation for training, assessment, and program evaluation are discussed below.

First, it provides a mechanism to guide learning through systematic, developmental feedback.[4,5] To meaningfully reflect on and integrate simulation-based learning experiences, learners must be provided with explicit, constructive feedback detailing their current performance levels and strategies for improvement.[6] Team members need to understand their performance level relative to expectations or standards before they can address how to improve.

Second, team performance evaluation enables summative assessment; that is, it allows trainers and team members themselves to obtain a snapshot of a team’s development at a particular time. Such assessments may help determine whether trainees possess the requisite team competencies for effective on the job performance upon the completion of training. Summative assessment may also be used to match current trainee skill mastery level to training objectives in order to create a more targeted and efficient training program. For example, team members may complete an initial simulation scenario to determine those team competencies most in need of refinement. In this light, team performance evaluation provides a mechanism for “individualizing” training to particular teams or constellations of potential team members, resulting in a more focused use of training time.

Third, by defining key behavioral and outcome criteria, evaluation provides a mechanism for SBT curriculum validation. Validating a curriculum answers the question, “does this training work?”.[7] Evaluation is the mechanism for determining the degree to which trainees apply knowledge, skills, and attitudes learned in training to similar situations (i.e., routine transfer) and/or to novel problems and new situations (i.e., adaptive transfer). In this way, team performance evaluation in simulation scenarios can serve the purpose of understanding the general level of team functioning within an organization in a controlled and systematic manner.

### What to measure?

While is it clear the evaluation is a vital component of SBTT, the question then arises what to measure? By definition, teams are composed of individuals, often having heterogeneous knowledge, skills, and attitudes, working interdependently to achieve a shared goal.[8] To achieve this goal, they must coordinate, communicate, and cooperate, each individual dynamically adjusting their own effort and input to the group based upon the effort and input of fellow team members.[9] Team performance outcomes are, thus, the synergistic product of these multiple inputs. Therefore, evaluation must not only capture the final outcome of team performance, but also how the team achieved these outcomes. To comprehensively evaluate team performance, training designers must employ multiple measurements, which capture the behavioral, cognitive, and attitudinal components of performance at the team level. This also means capturing diagnostic information on individual team member roles in order to provide targeted corrective feedback. While evaluation is not focused specifically on individual team members, providing role-specific feedback helps individual members on how to operate effectively within the specific team roles as well as helps to develop important teamwork competencies such as communication. In sum, the primary content of a team performance evaluation tool in SBT should be the teamwork competencies targeted for training: the knowledge, skills, and attitudes underlying effective teamwork.[10]

### How to evaluate team performance?

As noted earlier, fully capturing the range of team processes which comprise team performance involves multiple approaches to measurement. One pitfall of many attempts to evaluate team performance involves measuring what is easy to measure as opposed to what is meaningful to measure. For example, many early SBT studies only measured trainee reactions to the training program: did trainees like it, find it useful, and so forth. However, reactions to training have demonstrated relatively weak relationships to other, arguably, more important outcomes such as learning and transfer of learned KSAs to the daily work environment.[11] For example, training which includes difficulty and uncertainty may initially be rated as less enjoyable by trainees, especially those with a performance orientation, who strive to demonstrate their present level of skills.[12,13] However, more challenging training scenarios enable trainees to practice more advanced teamwork competencies under more realistic clinical conditions. Additionally, important, but broad, indicators of patient safety and quality (e.g., adjusted mortality rates) may not be sensitive to the components of team performance that impact patent outcomes.[14] Therefore, the methods and metrics used for team performance evaluation must be designed to capture both team process and outcomes.

Tools for team performance measurement and evaluation can be organized into three overarching categories: observational rating scales, team self-assessment, and the event-based approach to training and measurement. Observational rating scales are standardized measurement protocols, which train observers to record and rate observable team behavior.[15,16] Teamwork rating scales may employ traditional behaviorally anchored rating scales (BARS) or other graphical observational scales. Additionally, they may provide ratings of each dimension of teamwork, a global rating of teamwork, or both. Rating scales developed for rating team performance in healthcare settings have included global Likert-scale ratings (e.g., Mayo High Performance Teamwork Scale[17] and BARS scales),[18] as well as approaches which capture both the quantity and quality of teamwork behaviors (e.g., CATS[19]). Observational methods, of course, have weaknesses due to limitations and biases of individual raters. For example, while early research suggested that raters can rate validly up to seven behavioral dimensions at a time,[20] more recent suggestions are for limiting the number of behavioral dimensions to be rated to three to four.[21] Additionally, raters must be able to observe and rate behavior within the rich situational contexts of clinical simulations.

Event-based training and assessment techniques (EBAT) were specifically designed to deal with the complexities of rating performance during complex simulation scenarios.[22–24] In the EBAT approach, critical events which provide opportunities to perform key teamwork competencies are combined and embedded into relevant contextualized scenarios. Critical events can be routine or novel events which occur at a pre-determined point during the course of the simulation. Developing scenarios based upon critical events create both a script and timeline for the scenario, which helps raters to know explicitly what behaviors should occur, approximately when they should occur, and also provides a clear way to organize rating forms. This approach was originally developed to rate team performance during complex military simulation scenarios;[22] however, it has successfully been leveraged for ratings of teamwork during resident medical training.[24] A more detailed review of observational scales used in healthcare is presented later in this article.

While observational measures capture the behaviors teams actually engage in, observers cannot assess the implicit components of teamwork, including team cognition and implicit communication. To capture these elements of team performance, observational measures can be combined with self-assessment measures completed by team members. While self-report measures also have limitations, they offer a means for assessing unobservable components of team performance, which are no less important than observable components. For example, Sexton and colleagues[25] developed a component of their Operating Room Management Questionnaire to specifically capture team-member assessment of teamwork, during their last surgical case.

Different approaches to evaluating teamwork in simulations have associated strengths and weaknesses. Consequently, a combined approach leveraging best practices derived from the science of teamwork and human performance measurement can help to obtain a more complete picture of the complex phenomena, that is, team performance. The next section presents eight best practices for team performance evaluation based on a review of techniques currently being used in healthcare and other complex team environments.

## BEST PRACTICES

There is no “one size fits all” approach to measure team performance. As reviewed above, differences in purpose and content of measurement (e.g., the team competencies being trained or assessed) as well as the clinical context require different approaches. However, the fundamentals of team performance measurement and evaluation provide guidance development or selection and modification of team performance measurement tools for a given set of conditions. This section integrates recently advanced best practices in the measurement of team performance in SBT for healthcare[24] and provides practical tips for implementation. Table 1 provides a summary of these best practices, rationales for each, as well as practical tips to guide the development of measurement tools suited for a specific context. Each of these best practices is described below.

**Table 1:** Best practices in team performance measurement for SBT for healthcare, description and tips (adapted from Rosen *et al*.[30])

Best practice	Description/rationale	Tips for implementation
The content of a measurement tool should be driven by theoretically and empirically rooted competencies of teamwork	There is a well-developed science of team performance and a growing literature specific to healthcare domains; competency models should be developed based on the best evidence available	Is there a “consensus” model of teamwork in your clinical domain? If so, use it; if not, work from more general models of teamwork[6]
Measures should be linked to specific learning objectives	In SBT, measurement is one component of a broader system; to be maximally effective, all components of this system need to be aligned; the content of a measurement tool needs to capture the competencies specified in learning objectives	Design measurement tools in parallel with practice activities Be explicit about what is being trained for a given activity; what is being trained is what should be measured
Capture multiple levels of performance	The overall performance of a team involves individual level technical performance as well as teamwork behaviors; measurement tools must make distinctions between these two things in order to facilitate the provision of feedback at the appropriate level	Create opportunities to perform teamwork behaviors that are not linked to team members’ level of clinical knowledge or skill
		If possible, reduce the level of technical complexity in a scenario when training teamwork behaviors
Use scenario events to anchor measurement opportunities	Scenario events represent opportunities to perform a specific teamwork behavior; by focusing on these pre-defined points in the scenario where a targeted teamwork competency should be practiced, observers can focus their attention on critical aspects of the scenario	Create measurement tools with a temporal flow matched to the scenario to help structure observations
Focus measurement on observable team behaviors, and the *processes* of performance, not just the outcomes	Knowing the outcome of a team’s performance is insufficient for guiding improvement; knowledge about how the team reached that outcome (i.e., the processes of performance) is necessary	Keep measurement at a fine level of granularity so that it affords feedback on specific behaviors
		Avoid ratings that require summation over time or team members (e.g., the quality of communication across all team members throughout the entire scenario) as they provide poor support for feedback
Focus on “diagnosing” performance	In a practice-based learning activity, capturing what happened is important, but understanding *why* a team performed the way it did is necessary to guide feedback; understanding the underlying causes of performance is important for learning	Provide multiple opportunities to perform a targeted teamwork behavior
		Map out possible responses to a specific scenario event and reasons different “behavioral paths” would be taken
Train and monitor observers	The reliability and validity of a measurement tool is not a property of the tool itself, but how that tool is used; observers need to be trained so that they are “on the same” page in terms of expectations for performance	Use a scoring guide with definitions and examples of different types and levels of teamwork behaviors
		Use videotaped sessions of previous simulation exercises to train observers and assess the reliability of observer ratings
		Monitor reliability over time to avoid rater drift
Use measurement to support post-session debriefs and remediation	The real value of measurement is in driving learning and change in a systematic way; to do this, it needs to support the process of facilitated debriefs	Help teams with the *description* phase of debrief: coming to agreement on what happened during a scenario
		Help teams with the *analysis* phase of debrief: understanding what went well and what needs improvement

SBT: SIMULATION-BASED TRAINING

### Best Practice #1 – The content of a measurement tool should be driven by theoretically and empirically rooted competencies of teamwork

An initial and critical step in designing measurement tools for SBT for teams is to clearly define the content,[24] that is, the specific *teamwork* knowledge, skills, and attitudes that need to be measured and assessed. This content should be based in the best available evidence on the important characteristics of teamwork.[2,10] Ideally, the competency model is rooted in the specific context of the target clinical area; however, most clinical specialties have not developed consensus teamwork competency models to date. Therefore, adapting more general teamwork skills (e.g., basic communication, leadership, back-up behavior, etc.) is the primary option for training and evaluating teamwork skills in healthcare.

### Best Practice #2 – Measures should be linked to specific learning objectives

In training, measurement drives the provision of feedback. Therefore, the measures should have explicit and tight connections to the learning objectives—the educational goals for a given learning activity.[26] The content of the measurement tool (i.e., the “what” that is being measured) should be consistent with what is being trained.

### Best Practice #3 – Capture multiple levels of performance

It is important for measurement tools in SBTT to discriminate between the individual and team levels of performance. This can be a challenging goal to meet as team and technical performance are frequently intertwined. However, performance measures should distinguish as much as possible between individual-level performance and team-level performance, no matter what the purpose of evaluation. Separating these aspects of performance is critical for both formative assessments (e.g., is there a deficiency in an individual’s technical ability, or is there a problem with how the team works together?) as well as for generating feedback for learning in simulated environments.[27]

### Best Practice #4 – Use scenario events to anchor measurement opportunities

Team training scenarios can be fast-paced and complex. With many learners engaged in individual and team performance, a team-based practice activity can quickly overwhelm an observer’s limited attention and ability to accurately score performance. However, the control over scenario events in SBT affords a great advantage for measuring team performance (i.e., predictability of events requiring a teamwork response), which reduces the burden on observers. Specifically, observers can focus their attention on pre-determined critical events—circumstances in a scenario that require targeted teamwork skills to manage effectively.[22]

### Best Practice #5 – Focus measurement on observable team behaviors and the processes of performance, not just the outcomes

Emphasizing observable teamwork behaviors has two main advantages. First, creating and maintaining inter-rater reliability is simplified when measuring objective and directly observable behaviors, when compared to more abstract team concepts (e.g., team climate) that require observers to make judgments beyond what they are actually seeing. Second, measuring observable behaviors can provide team members with actionable guidance for improvement, that is, behaviors they can use in the future to improve performance.

### Best Practice #6 – Focus on “diagnosing” performance

Performance diagnosis entails uncovering the reasons by a team performed as they did—on identifying the underlying knowledge, skill, or attitude competencies that contributed to effective and ineffective team performance. This is a valuable approach to measurement in not only understanding the reasons why a team performed the way it did but also for providing the appropriate corrective feedback or remediation.[4]

### Best Practice #7 – Train and monitor observers

Measurement tools are only as good as the quality of data they produce. For observation-based measurements, the quality of the data relies not just on the tool, but the observer as well. For this reason, training observers is critical. Training can be facilitated with the use of scoring guides where the different types and levels of teamwork behaviors are clearly articulate. This type of guide and associated training helps to keep all observers and trainers on the same page in terms of expectations for team performance.

### Best Practice #8 – Use measurement to support post-session debriefs and remediation

Feedback drives learning, and in SBTT, facilitated debriefs are the primary method of delivering feedback.[28] Here, teams are expected to actively discuss what happened during the scenario and how this performance relates to standards. Debrief facilitators can use performance measurement tools to structure their observations and guide the team’s discussion to aspects of team performance that need reinforcement or correction.[29]

## TOOLS FOR OBSERVATIONAL METHODS IN SIMULATION-BASED TEAM TRAINING

Observation-based methods have been and remain the primary means of capturing team performance. This section provides an overview of four of the predominate tools available in the literature to date. These form different domains and are not necessarily designed for the simulation environment. However, an examination of these tools in light of the best practices for SBTT described above is useful for understanding what makes a good performance measurement tool in a team training environment. Table 2 provides a summary of each tool, and the following section describes the types of choices in content and method, which need to be made when developing or adapting a tool for the purposes of training.

**Table 2:** Overview of four observation-based team performance evaluation tools

Tool	Content	Scoring	Temporal organization
UT BAMF	Information sharing, inquiry, assertion, sharing intentions, teaching, evaluation of plans, workload management, vigilance/environmental awareness, overall teamwork, and leadership	Each behavior is scored in terms of observability and frequency of that behavior; ratings are made on 4 point scales	Raters are required to summate their judgments across an entire session (i.e., observability of a behavior throughout an observation period) and provide global frequency estimates for the session as a whole
ANTS	High level teamwork skills: task management, team working, situation awareness, and decision making	The presence of absence of behaviors is scored and then the quality of the behavior is rated on a 4-point scale	Teamwork ratings are summated across the entire session (e.g., the overall quality of task management) instead of capturing specific instances of good and poor task management
OTAS	High level teamwork skills: communication, leadership, coordination, monitoring, and cooperation	Teamwork behaviors are rated on a 7-point scale of quality	Ratings are organized into three stages: pre-operative, intraoperative, and post-operative
CATS	Coordination, cooperation, situational awareness, and communication	The frequency and quality of behaviors are captured by tallying the instances of a good teamwork behavior, a teamwork behavior performed but in need of improvement, and an instance where a teamwork behavior was expected but not observed	Ratings capture individual instance of behaviors, but these instances are not tied to specific events (i.e., they are tick marks on a sheet), so may be difficult to use in a debrief for calling out a particular behavior in a situation

First, the University of Texas Behavioral Marker Audit Form (UT BMAF)[31] was derived from team training methodologies utilized in aviation [i.e., the Line Operations Safety Audit (LOSA), an observational tool that assesses the behaviors of flight crews]. The UT BMAF is a one-page behavioral rating scale with three sections (i.e., event demographics, threats to patient care, and behavioral) designed to evaluate teamwork behaviors performed during neonatal resuscitations. In addition to incorporating LOSA, focus groups, surveys, and video recordings resulted in 10 behavioral markers: information sharing, inquiry, assertion, intentions shared, teaching, evaluation of plans, workload management, vigilance/environmental awareness, teamwork overall, and leadership. Each behavior is rated on two scales: observability (i.e., how well a behavior could be observed) and frequency (i.e., how consistently behaviors were exhibited).

Second, the Anesthetists’ Non-Technical Skills (ANTS) System was developed in collaboration between industrial psychologists and consultant anesthetists in a 4-year research project attempting to develop a taxonomy for structured observations of anesthetists. The ANTS System is a behavioral marking system which describes observable non-technical skills associated with good anesthetic practice.[32] The ANTS System is designed as a hierarchy, with 4 higher level skill categories (i.e., task management, team working, situation awareness, and decision making) and 15 lower level skill elements within these four dimensions. Each element is further defined with markers of good and poor behaviors to facilitate the observer in determining the presence/absence of the higher skill elements. Ratings of both the higher and lower level skill categories are made on a four-point scale to describe the quality of the observed performance (1-Poor, 2-Marginal, 3-Acceptable, 4-Good). The rating form also contains areas to note observations on performance and debriefing notes (which is encouraged after every observation session). Furthermore, the ANTS system has been empirically tested, with acceptable levels of validity, reliability, and usability, using a method previously used for NOTECHS,[33] a system from aviation.

Third, the Observational Teamwork Assessment of Surgery (OTAS)[34] was developed to evaluate technical and interpersonal skills in surgery teams. OTAS measures two main facets (taskwork and teamwork) of the surgical process in three stages: pre-operative, intra-operative, and post-operative. The first component, the clinical checklist, is completed by a surgical expert and is subdivided into categories associated with patient, equipment/provisions, or communications. Items are rated dichotomously (i.e., as “yes” or “no”) indicating whether or not the behavior was appropriately completed. The second component assesses teamwork behaviors (communication, leadership, coordination, monitoring, and cooperation) and is preferably rated by an expert researcher who scores behaviors on a 7-point Likert scale.

Fourth, derived from LOSA, ANTS, and OTAS, the Communication and Teamwork Skills[19] Assessment is designed to measure communication and teamwork behaviors of medical professionals in multiple clinical settings. Ratings are made on four clusters of behavioral markers (i.e., coordination, cooperation, situational awareness, and communication), and teams are scored both on frequency and quality. Rating the frequency of behaviors provides evidence as to how often behaviors are occurring; however, there is no indication of whether or not behaviors are being performed correctly. Thus, behaviors are also rated by quality, which can assist clinicians in determining the specific areas of improvements. Furthermore, scores can be aggregated to an overall score, or they can be parsed out to specific, individual behaviors. The benefit of providing several individual and team scoring techniques allows clinicians to evaluate behaviors on multiple levels, which offers a thorough assessment of both the team as a whole and the individual team members.

## IMPLICATIONS

The tools reviewed above represent some of the best instruments available in the published literature. However, these tools were not all developed specifically for use in a training environment. Therefore, some of the characteristics of these tools do not map onto the best practices described above. This section highlights some of these considerations for developing tools for SBTT.

Issues of scoring and temporal organization are important to meet the demands of guiding learning in simulation (see best practices 4–6). Many of the tools reviewed above require observers to summate their ratings across time by providing for example, an overall rating of the quality of a specific teamwork behavior during an entire observation session. This is satisfactory if the goal is purely assessment; however, in order to provide meaningful feedback, more specific aspects of the team’s appropriate and inappropriate use (or lack of use) of a behavior are more useful. At best, a global rating can provide team members with an indication of whether or not there is a problem with their performance, but it provides very little information about how to improve their performance. Additionally, the content of the measurement tool is critical if it is to support learning (see best practices 1, 2, 6, and 8). Ratings on very high level or abstract “dimensions of teamwork” (e.g., overall teamwork) do not provide the specificity necessary to guide learners in their development. However, capturing a particular example of a specific teamwork behavior (e.g., was there a closed-loop communication when a medication order was called out?) can feed directly into a learning point on post-performance debrief.

As described earlier, EBAT[22,24] is a method for developing measurement tools that meet all of the best practices outlined above. The challenge with these tools is that each is scenario specific. However, the added investment in developing measurement tools for each training scenario pays dividends in the quality of measurement as well as the quality of the training. While general methods have been outlined for doing the EBAT process, future work is needed to develop tools to structure and facilitate this process for practitioners.[35]

## CONCLUSION

It is an exciting time to be involved in the development of clinical practitioners. New technology and new thinking on what is important for effectiveness have created an opportunity to improve the quality and safety of patient care with inventive strategies. This article has provided a brief review of the concepts and methods of team performance measurement in SBT, one component of a broader initiative to transform healthcare into a high-reliability organization. This is a new area for healthcare, and there is much work to do before good practices are widely adopted. However, the types of methods and tools discussed in this review can contribute to getting the most out of SBTT.
